# Age Hardening Characteristics of an Ultra-Low Carbon Cu Bearing Steel

**DOI:** 10.3390/ma13184104

**Published:** 2020-09-16

**Authors:** Mingxue Sun, Yang Xu

**Affiliations:** School of Mechanical and Automotive Engineering, Qingdao University of Technology, Qingdao 266520, China; xuyang@qut.edu.cn

**Keywords:** aging, Cu precipitation, reverted austenite, transmission electron microscopy, structure-property relationship

## Abstract

We studied the influence of aging temperature on microstructure and mechanical properties in an ultra-low carbon Cu bearing steel in the present study. During the aging process, a continuous recovery of matrix associated with formation and growth of Cu precipitates could be observed during aging processes, exerting significant effects on the mechanical properties of the steel. At aging temperature below 600 °C, the mechanical properties were dominated by the precipitation strengthening effect, leading to excessive matrix strengthening and poor low-temperature toughness. Conversely, steel aged at temperatures above 650 °C exhibited an extraordinary improvement in toughness at the expense of strength, which can be attributed to the synergistic effects of softening matrix, coarsened Cu precipitates and formation of reverted austenite. After aging at 650 °C, reverted austenite formed at the lath boundaries. Increasing the aging temperature to 700 °C lowered the thermal stability of reverted austenite, consequently, the reverted austenite was partially transformed to fresh martensite. After aging at 650 °C for 0.5 h, the mechanical properties were optimized as follows—yield strength = 854 MPa, tensile strength = 990 MPa, elongation = 19.8% and Charpy impact energy = 132 J at −80 °C.

## 1. Introduction

Ultra-low carbon Cu-bearing steels, such as ASTM A710, HSLA-80, HSLA-100 and NUCu-140, have been extensively developed over recent decades [1,2,3,4]. In practical applications, these steels require high strength, good low temperature toughness and excellent weldability. Because the C additive decreases the weldability and impact toughness of steel, decreasing the C content without adversely affecting the mechanical properties has been a significant strategy in steel development. To compensate for the decreased strength caused by the lowered C concentration, these steels are reinforced with 1–2 wt % Cu, which provides enormous precipitation strengthening without seriously deteriorating the weldability. Therefore, understanding the Cu precipitation mechanism is crucial for improving the mechanical properties of Cu-bearing steels. The evolutions of precipitation morphologies [5,6], precipitation strengthening [7,8], crystal structure [9,10,11] and chemical composition [12,13,14,15] of Cu particles have been extensively studied. The early stage of Cu precipitation is characterized by coherent body-centered cubic (bcc) particles with small size (<5 nm). During the aging process, the bcc structure transforms to a 9R structure and finally to a stable face-centered cubic (fcc) structure. The crystal-structure evolution is accompanied by a change in the elemental composition of the steel. The solute elements (Ni, Mn and Al) partition into the cores during the initial precipitation stage and then segregate at the interfaces between the precipitates and matrix [16].

The bcc structure is stable up to the aging peak. At the aging peak, the strength is maximized but the toughness is poor. The low temperature toughness is improved at the cost of reducing the strength in the over-aged stage. Therefore, these steels are usually developed in the over-aged state, during which the softening matrix and coarsening Cu precipitates provide an excellent combination of strength and toughness [3,17,18,19]. 

Many reports found that the reverted austenite can be obtained during the intercritical heat treatment. When tempered at a temperature slightly above A_c1_, a solute-rich austenite phase formed and retained to the room temperature because of the high thermal stability. As is well known, introducing reverted austenite with high stability improves the low temperature toughness of steels [20,21,22]. Chen et al. [21] proved that the thermal stability of reverted austenite is mainly determined by its chemical composition, size and morphology in a low carbon medium Mn steel. Excellent mechanical properties (strength, ductility, low temperature toughness and strain hardening capacity) can be obtained by controlling the thermal stability and volume fraction of reverted austenite. However, previous reports have mainly focused on medium Mn and/or high Ni steels, whereas ultra-low carbon Cu-bearing steels have been largely ignored. Utilizing reverted austenite with high stability is of technical importance in ultra-low carbon Cu-bearing steels.

The present study offers a new strategy for toughening ultra-low carbon Cu bearing steel. The microstructural evolution, Cu precipitation behaviors and resultant mechanical properties are fully characterized by scanning electron microscopy (SEM), transmission electron microscopy (TEM), tensile testing and impact testing. Finally, an appropriate process that achieves good mechanical properties is suggested.

## 2. Materials and Methods

**Figure 1:**
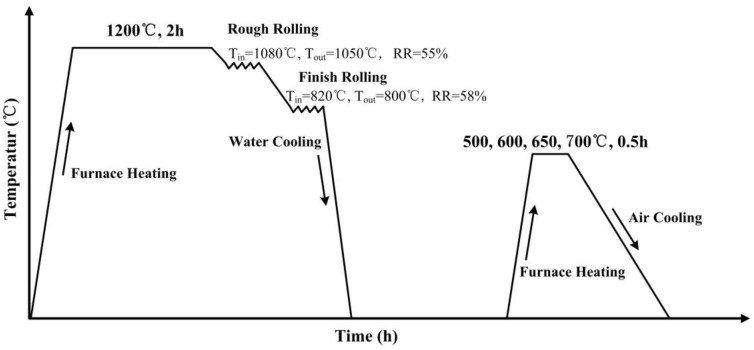
Schematic of the processing conditions of ultra-low carbon Cu-bearing steel.

The chemical composition of the test steel was designed as follows: 0.02 wt % C, 0.2 wt % Si, 1.5 wt % Mn, 1.5 wt % Cu, 3.5 wt % Ni, 0.5 wt % Cr, 0.5 wt % Mo and 0.03 wt % Nb. The carbon content was decreased to 0.02 wt % to improve the weldability and 1.5 wt % Mn and 3.5 wt % Ni were added to obtain reverted austenite. The steel was melted in a laboratory vacuum furnace, followed by forging and cutting into billets with dimensions of 80 mm × 80 mm × 100 mm. Figure 1 is a schematic of the processing conditions. The billets were austenitized at 1200 °C for 2 h and then hot rolled to a final thickness of 15 mm using a two-high pilot mill (State key laboratory of rolling and automation, Shenyang, China). The rough rolling was conducted in the temperature range 1050 °C–1080 °C, with a rolling reduction (RR) of 55%. In the non-recrystallization region of austenite, the finish rolling temperature was varied between 800 and 820 °C and the RR was 58%. The hot rolled plates were subsequently water cooled to room temperature at a cooling rate of ~40 °C/s. After the rolling stage, the plates were aged at 500, 600, 650 and 700 °C for 0.5 h in a RX3-24-10 box-type electrical‒resistance furnace (SYTYDL, Shenyang, China) and then air cooled to room temperature.

Specimens for tensile and Charpy impact tests were cut from the plates along the rolling direction (RD). Longitudinal round tensile specimens of diameter 10 mm and gauge length 50 mm were machined according to the ASTM E8M standard [23]. Tensile testing was conducted in a CMT-5105 testing machine (MTS, Shenzhen, China) with a crosshead speed of 3 mm/min. The Charpy impact tests were carried out in an Instron 9250 HV Drop Weight Tester (Instron Corporation, Norwood, MA, USA) at the temperature of −80 °C. The specimens were sized 10 mm × 10 mm × 55 mm and prepared in accordance with the ASTM E23 standard. Microstructural examination along the cross section defined by transverse direction (TD) and normal direction (ND) was conducted in a FEI Quanta 600 SEM (FEI, Hillsboro, OR, USA). These samples were mechanically polished and then etched in 4% nital solution. TEM and scanning transmission electron microscopy (STEM) analyses were examined in a FEI Tecnai G2 F20 TEM (FEI, Hillsboro, OR, USA) equipped with an energy dispersive X-ray spectrometer (EDX). Thin foils (thickness ~50 μm) were prepared using sandpapers. These foils were then electropolished in a solution of 6.25% deionized water, 12.50% perchloric acid and 81.25 % ethyl alcohol.

## 3. Results

### 3.1. Mechanical Properties

**Figure 2:**
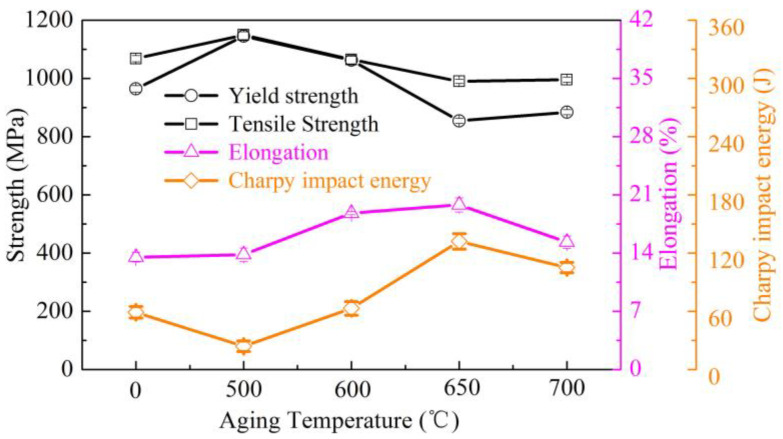
Variation of the mechanical properties with aging temperature of the tested steel.

**Figure 3:**
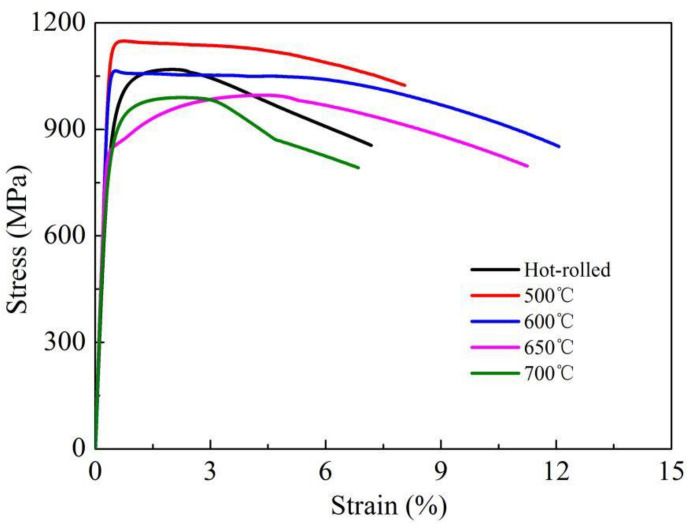
Engineering stress vs. engineering strain curves of the steels aged at different temperatures.

Figure 2 represents the mechanical properties of the tested steel under the water-cooled condition and after aging at different temperatures. The typical engineering stress versus engineering strain curves are shown in Figure 3.

Although the water-cooled steel achieved high yield and tensile strengths (964 and 1068 MPa, respectively), the elongation (13.5%) was poor and the Charpy impact energy at −80 °C was only 59 J. The mechanical properties were notably changed after the aging process. After aging at 500 °C, the steel reached its peak strength but the Charpy impact energy at −80 °C was lowest. At aging temperatures above 500 °C, the toughness was improved at the expense of strength. The elongation and Charpy impact energy at −80 °C showed the highest values of 19.8% and 132 J at aging temperature of 650 °C. However, at this temperature, the yield and tensile strengths decreased to 854 and 990 MPa, respectively. The strength slightly increased at 700 °C but with some loss of the Charpy impact energy (to 105 J) and elongation (to 15.3%).

### 3.2. Microstructural Characterization

**Figure 4:**
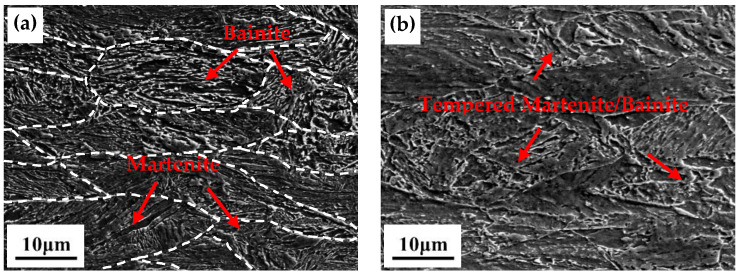

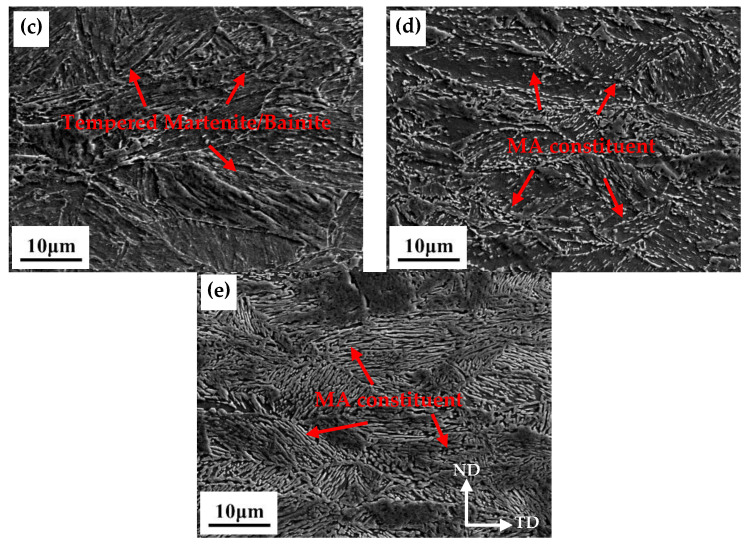
The scanning electron microscopy (SEM) micrographs of the tested steel under different aging conditions: (**a**) water cooling only, (**b**) aging at 500 °C, (**c**) aging at 600 °C, (**d**) aging at 650 °C, (**e**) aging at 700 °C.

Figure 4 shows the SEM micrographs of the test steels processed under different conditions. As shown in Figure 4a, the prior-austenite grains were severely deformed and elongated parallel to the transverse direction. The microstructure reveals a mixed structure of lath bainite/martensite, where lath bainite can be revealed with the characteristic of carbides between laths. The morphologies were noticeably changed after aging at different temperatures. Microstructural recovery was evidenced during the aging process. At aging temperatures of 500 °C and 600 °C, the tempered martenite/bainite with the lath-like microstructure can be observed. Due to the resolving of martenite, tempered martenite and bainite were almost indistinguishable, as shown in Figure 4b,c. When the aging temperature increased to 650 °C, tiny island-like structures appeared in the tempered bainite/martenite structure. These structures were identified as the martenite austenite (MA) constituent. During the heating and holding processes, composition fluctuations caused the nucleation of austenite grains on some defects. Under air cooling, these austenite grains were either retained or transformed into martensite. The volume fraction of MA constituents increased at an aging temperature of 700 °C and the morphology changed from island-like to lath-shaped (Figure 4e).

**Figure 5:**
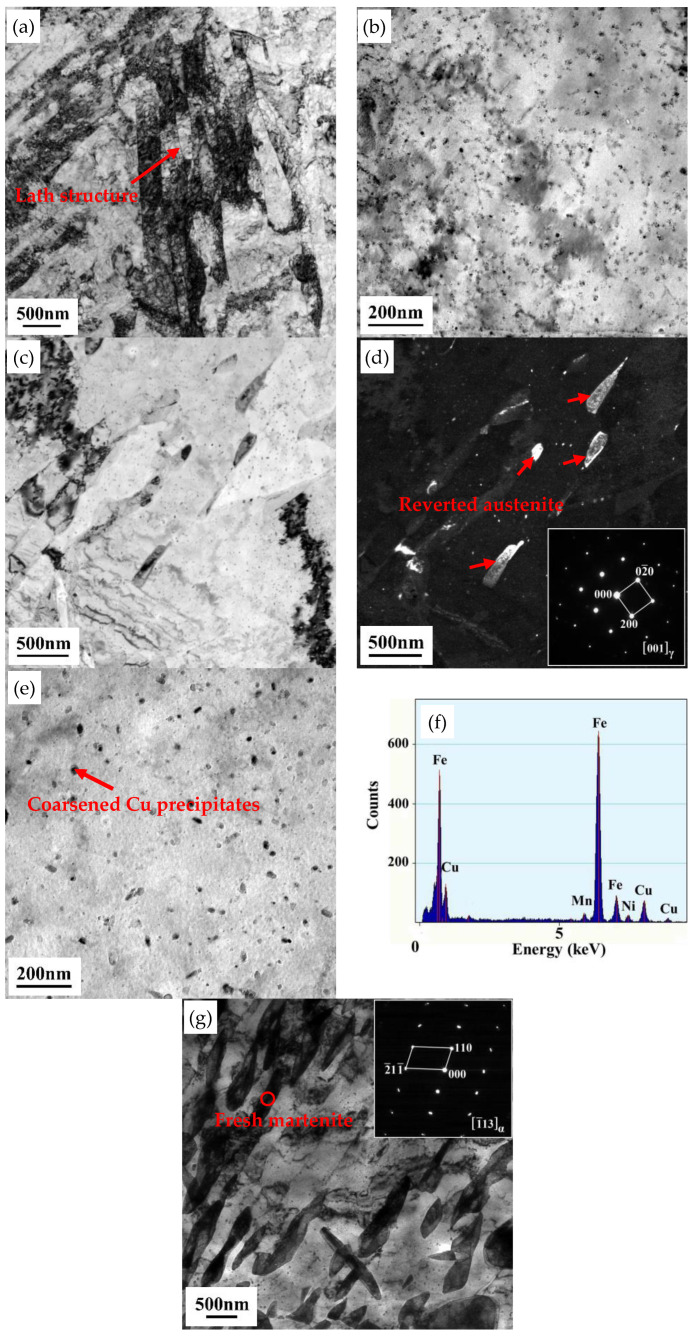
Transmission electron microscopy (TEM) micrographs of the tested steel: (**a**) partially recovered lath structure after aging at 600 °C, (**b**) uniformly distributed precipitates in the matrix after aging at 600 °C, (**c**) bright-field (BF) image after aging at 650 °C, (**d**) dark-field (DF) image of reverted austenite and its selected area electron diffraction (SAED) pattern (inset) after aging at 650 °C, (**e**) coarsened precipitates after aging at 650 °C, (**f**) composition determined by energy dispersive X-ray spectrometer (EDS) for the arrowed particle in (**e**), (**g**) BF image showing reverted austenite partially transformed to martenite after aging at 700 °C and its SAED pattern (inset) taken from the region indicated by a red circle.

**Figure 6:**
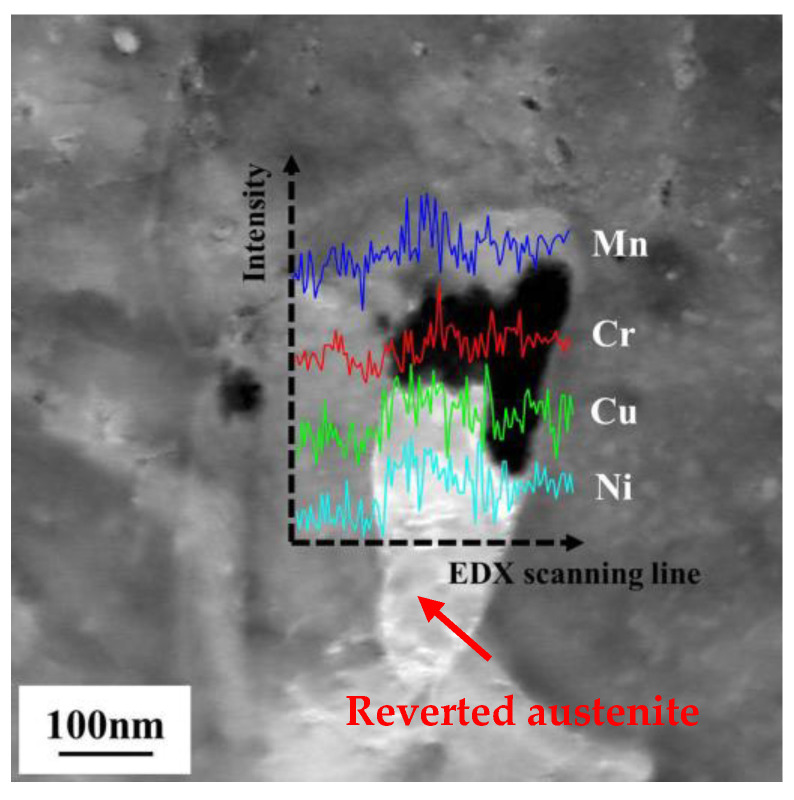
Scanning transmission electron microscopy-high angle annular dark field (STEM-HAADF) micrograph of the reverted austenite in the sample aged at 650 °C and EDX line-scanning results along the horizontal dark arrow line.

Figure 5 presents the TEM micrographs of the tested steel after aging at 600, 650 and 700 °C for 0.5 h. After aging at 600 °C, the lath structure was partially recovered and high-density dislocations appeared (Figure 4a). The lath spacing was measured as 0.2–0.4 μm. The laths contained uniformly distributed precipitates (approximate diameter 10 nm; Figure 5b), which were confirmed as Cu and Nb(C, N) in previous studies [3,24,25,26]. Dhua et al. [25] and Mujahid et al. [26] reported a large number of Cu precipitates and few Nb(C, N) precipitates in these ultra-low carbon Cu-bearing steels. As the Nb(C, N) precipitates were mainly formed during the thermomechanical process, they should not significantly affect the aging behaviors. After aging at 650 °C, the structure was largely recovered, showing a lower dislocation density and coarsened precipitates. In particular, selected area electron diffraction (SAED) analysis revealed the formation of lath-shaped austenite at the lath boundaries. These formations are depicted in the bright-field (BF) and dark-field (DF) images in Figure 5c,d, respectively. The austenite growth was mainly parallel to the lath boundaries. This newly formed austenite was identified as reverted austenite [25,26]. The precipitates were rod-like and their diameters grew to approximately 20–30 nm. The number and density of the precipitates also decreased, as shown in Figure 5e. In the EDS analysis, these coarsened precipitates were revealed as Cu-enriched particles (Figure 5f). At the highest aging temperature (700 °C), the reverted austenite increased in both size and volume fraction and was partially transformed to fresh martenite as demonstrated by the SAED pattern in Figure 5g. The reverted austenite obtained at aging temperature of 650 °C was further investigated by STEM analysis. Figure 6 shows the STEM-high angle annular dark field (STEM-HADDF) micrograph of the reverted austenite and EDX line-scanning results along the horizontal dark arrow line. The reverted austenite exhibited filmy morphology with the dimension of ~330 nm length and ~90 nm width. Mn, Cr, Cu and Ni elements were enriched in reverted austenite, suggesting that the partition of these elements occurred during the aging process.

## 4. Discussion

### 4.1. Effect of Processing on Microstructure

It has been confirmed that the maximum solubility of Cu in austenite is 2.1 wt % at the temperature of 850 °C [26]. Therefore, the austenitizing temperature is high enough to dissolve all Cu in this study. A supersaturated solid solution of Cu can be obtained during the water cooling process and the nano-sized Cu precipitates are formed during the aging condition. In numerous reports, the aging process in ultra-low carbon Cu-bearing steels is described as the recovery of the lath structure and the formation and coarsening of precipitates [3,18,19,20,24,25,26]. After aging at 600 °C, the lath structure was only partially recovered and the Cu precipitates were spherical. These Cu precipitates can effectively delay the matrix softening by sub-boundary pinning [25]. The matrix softening and growth of Cu precipitates increased with aging temperature. Therefore, at 650 °C, the lath structure showed a low dislocation density and the number and density of the Cu precipitates were lower than at 600 °C. In addition, newly formed reverted austenite was also observed. Reverted austenite was enriched in austenite-stabilizing elements, which stabilized the austenite during the air cooling process [26]. The thermal stability of reverted austenite is correlated with its composition, size and morphology. Increasing the aging temperature increases the volume fraction and dilutes the stabilizing elements in the reverted austenite, thereby reducing the thermal stability [21]. Hence, the reverted austenite transforms to fresh martensite at higher aging temperatures.

### 4.2. Effect of Processing on Mechanical Properties

In the present study, the hot rolled plates were water cooled at approximately 40 °C/s. The high strength of water cooled steel can be mainly attributed to the highly dislocated lath bainite/matenite structure. The lath structure obviously deteriorated the ductility and toughness of the steel. At aging temperatures below 600 °C, the steel derived high strength from the formation of numerous fine Cu precipitates, as similarly reported by Dhua et al. [25] and Mujahid et al. [26]. These Cu precipitates provide an additional strengthening effect at the expense of low temperature toughness. After aging at 650 °C, the strength was sharply decreased as the matrix continuously recovered and the Cu precipitates coarsened. In contrast, the ductility and low temperature toughness were remarkably improved. The TEM observation confirmed coarsening of the Cu precipitates and the formation of stable austenite at this aging temperature. Reverted austenite is known to improve the impact toughness by inhibiting crack propagation [27,28,29]. According to Panwar et al [30], small Cu precipitates cause high stress concentrations at the precipitate‒matrix phase interfaces. In the present study, the combined stress concentrations and high-density dislocation cells likely induced the nucleation of cracks and microvoids during the impact process. As a result, the Charpy impact energy largely reduced. The coarsened Cu precipitates formed in the over-aged stage help to arrest the propagation of cleavage cracks, thereby improving the toughness [30,31]. The co-existing recovery lath structure, coarsened Cu precipitates and newly formed reverted austenite remarkably improved the low-temperature toughness. Overall, the multiphase structure obtained at 650 °C provided excellent comprehensive mechanical properties. When the aging temperature increased to 700 °C, the thermal stability of the reverted austenite was decreased because the average contents of the austenite-stabilizing elements were lowered. This implies that the reverted austenite transformed to fresh martensite during air cooling, enhancing the strength of the steel but deteriorating its ductility and toughness.

The transformation behavior of the reverted austenite is very important, because it acts as an effective source of toughening. The composition of reverted austenite plays a dominate role on its thermal stability. During the austenite reverted transformation process, the austenite stabilizers diffuse to austenite from the matrix due to the different solid solubility between austenite and ferrite matrix. The reverted austenite becomes alloy-enriched and remains thermally stable upon cooling. The dilution of austenite stabilizers at higher aging temperature decreases the thermally stability of austenite, leading to a transformation from reverted austenite to fresh martenite [27,28,29]. Therefore, achieving stable reverted austenite is crucial for the toughening. In the present study, Mn, Ni, Cu and Cr elements enriched in reverted austenite, which made the austenite stable enough to remain at room temperature after aging at 650 °C. As reported in previous studies [3,17,18,19], the addition of Cu mainly contributed to the strength increment in the form of nano-sized Cu precipitates. The Charpy impact energy was promoted by the matrix recovery and coarsened Cu precipitates in over-aged condition. Different from these reports, it is obvious that Cu also provided toughening effects in the form of austenite stabilizer in this paper. By the corporation of the matrix recovery, coarsening of Cu precipitates and the formation of stable reverted austenite, the Charpy impact energy was fully improved when aged at 650 °C.

## 5. Conclusions

The main findings of the study are summarized below.

1. Water-cooled steel exhibited a lath bainite/martensite structure and the lath structure was continuously recovered during the aging process. The formation and coarsening of Cu precipitates increased with aging temperature.
2. After aging at 650 °C, the newly formed reverted austenite was sufficiently stable and was retained after air cooling. When the aging temperature increased to 700 °C, the thermal stability declined and the reverted austenite transformed to martenite.
3. The steel showed a typical aging behavior. The maximum yield and tensile strengths obtained at 500 °C were 1063 and 1065 MPa, respectively, along with the poor elongation of 13.8% and Charpy impact energy of 24 J. When the aging temperature increased to 650 °C, the yield strength and tensile strength were decreased by 291 and 159 MPa, respectively, while the Charpy impact energy was increased by 108 J.
4. After aging at 700 °C, due to the transformation from reverted austenite to fresh martenite, the yield and tensile strengths were slightly increased by 30 and 6 MPa, respectively but with some loss of the Charpy impact energy (by 27 J) and elongation (by 4.5%).
5. The impact toughness was improved at the expense of strength as the aging temperature increased. The best combination of strength and toughness was obtained after aging at 650 °C. These enhancements were associated with matrix recovery, lowering of the Cu precipitation strengthening effect and formation of reverted austenite.
